# Military Medical Heroism

**Published:** 1915-02-27

**Authors:** 


### The Hospital

The Workers' Newspaper of Administrative Medicine and Institutional Life, Administration, National Insurance and Health.

No. 1497, Vol. LVII. SATURDAY, FEBRUARY 27, 1915. PRICE ONE PENNY.

### MILITARY MEDICAL HEROISM.

It is impossible to record without pride not only the double Victoria Cross which, unique honour for valour, has been won by a medical man, but also the kindred distinctions earned by members of the Royal Army Medical Corps and the privates and other members of Red Cross or Field Ambulance units. The names contained in the Honours List published in last week's *Gazette* we record elsewhere; but the fact that medical men are mentioned in it at all suggests reflections on the nature of medical heroism in war-time, which are apt to be lost sight of in days when the lay Press so largely exists on manufactured sensations. We can well believe that nothing could be more distasteful to the recipients than the gush over their deeds to which the nation has been treated. The man who has been at the Front, seen the actual conditions, faced the terrible facts, knows that as a survivor of that experience personal laudation is as much out of place as sympathy at a funeral. The real truth will simply not bear talking about. We therefore do not propose to emulate these performances, but rather to consider in a sober spirit the striking limitation which the function of the medical officer on the battlefield necessarily imposes on him in reference to actions in which the active combatants very properly engage.

How real the medical officer's limitations are in the matter of valour, as usually conceived, can be simply shown from the fact that the Royal Army Medical Corps is present to preserve life, while the armed soldier is there to destroy it. Difference in function thus involves difference in the action which is proper to, and praiseworthy for, each. In the present Honours List one Victoria Cross has been granted to a man who is officially described as virtually storming a position by himself, while killing a number of Germans single-handed in the process. Medical V.C.s, on the other hand, have of course been granted for acts of bravery in risking life to save or succour wounded men. But, as was pointed out by the medical officer, whose recently published articles in The Hospital flashed such a vivid light on ambulance work in France, the medical officer's duty to save life includes the duty of taking precautions for his own safety. His first duty is self-control; his second to do everything possible for the wounded without unduly exposing himself. Thus the medical hero is one who realises the value of his life and services to the troops he serves; and also never forgets that while a soldier may perhaps be made in six months, it takes six years to make a doctor. The same principles guiding action in the field apply also to the lay volunteers in the Red Cross units. Bandaging and first-aid can be relatively quickly learned, it is true. Nevertheless, the trained man is a unit of value, and his primary duty is not to dare the risk of his own life, but to save the lives of his wounded comrades.

Thus honours for active and open valour are less within the ambition of the Medical Service than of any other branch of the Army. Moreover, the need for preventing recklessness among the medical men so early impressed itself by the losses entailed on them in the earlier days of the campaign, that we may rest assured that those now awarded distinctions have taken the measure of their function with true responsibility and self-control, and earned fame, indeed, as much by the risks which they have avoided as by those which they have equally bravely undergone. How large, in the light of this, then, become risks which it may be proper to undertake! Can anything bring home to us the appalling destructiveness of modern warfare better?

Let us not forget, however, that self-control is also needed at home, and that the nation could well show a little more of it. Talking recently with a Red Cross worker, lately returned wounded from the Front, he told us that one of the facts which forced itself on him after two months' experience was the far greater number of English than French wounded who suffered from shock after operations. He added also that a visit paid both to London and Paris, shortly after the declaration of war, showed the former far more excitable than the French city. We give for what they may be worth these personal impressions from the mind of one well situated to form a judgment. They serve to remind us of the criticisms of George Meredith and of another fine observer, which latter, a few years ago, remarked: "Twenty years of cheap reading have changed the English from the most stolid to the most hysterical nation in Europe." At all events, whatever the future brings, we cannot improve on the wise phrase in the Litany, "in all time of our tribulation, in all time of our wealth," grant us courage; and the type of courage that we all need is that, as the Honours List shows, soberly practised by the medical men at the Front.

